# Multiple Myeloma of the Central Nervous System: 13 Cases and Review of the Literature

**DOI:** 10.1155/2018/3970169

**Published:** 2018-04-23

**Authors:** Gergely Varga, Gábor Mikala, László Gopcsa, Zoltán Csukly, Sarolta Kollai, György Balázs, Tímár Botond, Nikolett Wohner, Laura Horváth, Gergely Szombath, Péter Farkas, Tamás Masszi

**Affiliations:** ^1^3rd Department of Internal Medicine, Semmelweis University, Budapest, Hungary; ^2^Department of Haematology and Stem Cell Transplantation, St. István and St. László Hospital, Budapest, Hungary; ^3^Department of Neurology, Semmelweis University, Budapest, Hungary; ^4^Heart and Vascular Center, Division of Diagnostic Imaging, Semmelweis University, Budapest, Hungary; ^5^1st Department of Pathology and Experimental Cancer Research, Semmelweis University, Budapest, Hungary

## Abstract

Central nervous system involvement is a rare complication of multiple myeloma with extremely poor prognosis as it usually fails to respond to therapy. We present 13 cases diagnosed at two centers in Budapest and review the current literature. The majority of our cases presented with high-risk features initially; two had plasma cell leukemia. Repeated genetic tests showed clonal evolution in 3 cases. Treatments varied according to the era, and efficacy was poor as generally reported in the literature. Only one patient is currently alive, with 3-month follow-up, and the patient responded to daratumumab-based treatment. Recent case reports show promising effectivity of pomalidomide and marizomib.

## 1. Introduction

Multiple myeloma (MM) typically presents with skeletal symptoms (back pain, vertebral fractures, and lytic lesions), cytopenias (anemia, thrombocytopenia, and neutropenia), renal failure, and infections related to immunoparesis [1]. Extramedullary propagation is a rare event with poor prognosis due to frequent treatment failure. Within this group, central nervous system (CNS) involvement is a particularly problematic complication with an even worse prognosis.

The treatment of MM developed a lot in recent years. From the early 2000s, first thalidomide and its analogues and then bortezomib and newer proteasome inhibitors were introduced, followed by anti-CD38 and anti-CS1 antibodies getting FDA and EMA approval most recently. As a result, the expected survival of MM increased to 5–10 years in the majority of patients [2]. The longer survival corresponds to more extensive disease evolution that sometimes results in previously unseen, more resistant, and more aggressive clones with features uncommon in historical cohorts. Normally myeloma cells need constant support from the bone marrow stromal cells; however, evolution of the disease sometimes leads to clones that can survive independently in extramedullary tissues. This is rare at presentation (7%) but becomes increasingly common at subsequent relapses in the form of organ involvement, soft tissue tumors, plasma cell leukemia, and rarely (less than 1%) CNS involvement, which is the focus of this paper [3]. Due to its rarity, clinical trials are missing in this field; therefore, most of our knowledge is derived from case reports and retrospective reviews. As a consequence, we do not have evidence-based treatment guidelines.

Based on a review by* Dispenzieri* and* Kyle,* intracranial plasmacytomas or myelomas can be classified into four groups: (1) those extending from the skull pressing inward, (2) those growing from the dura mater or the leptomeninges, (3) those arising from the mucous membranes of a nasopharyngeal plasmacytoma, and (4) intraparenchymal lesions without evidence of extension from any of these other three sites [4]. Leptomeningeal involvement is the most common form, often resulting in nerve root infiltration and cerebral nerve palsies.

## 2. Symptoms

Specific symptoms depend on the underlying abnormality. Headache, confusion, cerebral nerve palsy, and radiculopathy are the most common general symptoms at presentation. In case of intraparenchymal lesions, focal neurologic symptoms can sometimes occur together with somnolence or seizures. The diagnosis of CNS MM is based on imaging and/or cerebrospinal fluid (CSF) analysis, with stereotaxic brain biopsy, if applicable [5].

## 3. Imaging

**Figure 1:**
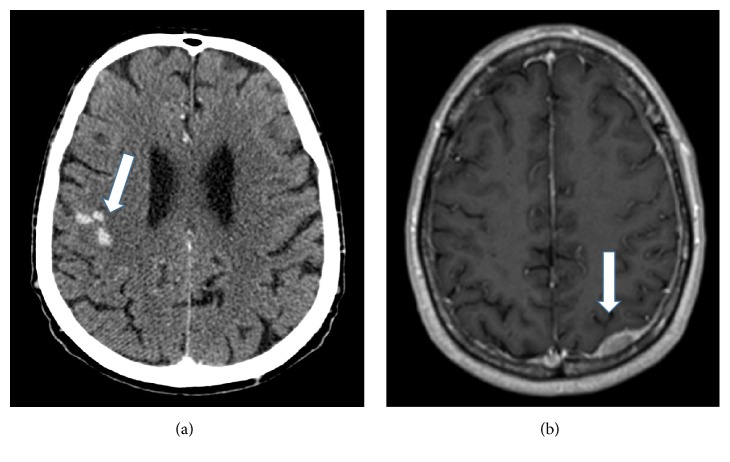
CT and MRI images of meningeal myeloma. (a) Contrast-enhanced CT; arrows: abnormally enhancing, leptomeningeal nodular lesions in the right frontal sulci (patient 4); (b) T1-weighted brain MRI after Gadolinium administration; arrows: abnormally enhancing dural lesion to be differentiated from subdural hemorrhage (patient 11).

**Figure 2:**
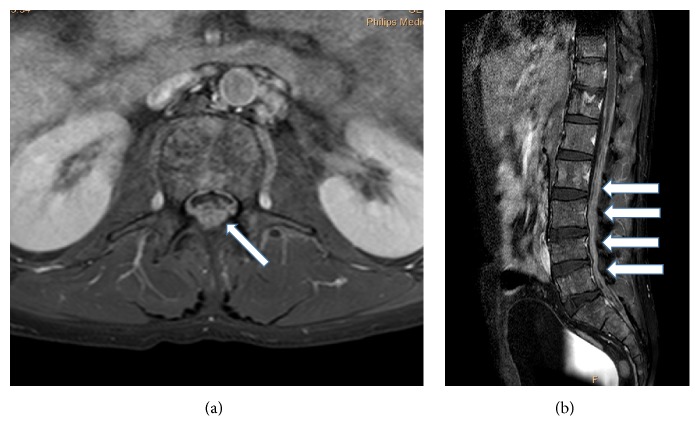
Gadolinium enhanced T1-weighted MRI images of leptomeningeal myeloma of the lumbosacral spine. (a) Axial and (b) sagittal image; arrows: contrast-enhanced myelomatous deposits (patient 11).

In suspected CNS MM, the first diagnostic step is usually contrast-enhanced MRI (head and/or whole spine). The sensitivity of MRI is over 90%: it can demonstrate bone-originated plasmacytomas, intraparenchymal tumorous lesions, or leptomeningeal contrast-enhanced deposits (Figures 1 and 2). In a review, the latter was the most common form, present in 70% of the cases [6]. CT can be diagnostic as well but with lower sensitivity (80%), and iodinated contrast material is often contraindicated in myeloma [3]. However, the abnormalities are not necessarily specific; for example, they can mimic subdural hemorrhage as in patient 11 in our series [6] (Figure 1(b)).

## 4. CSF Analysis

**Figure 3:**
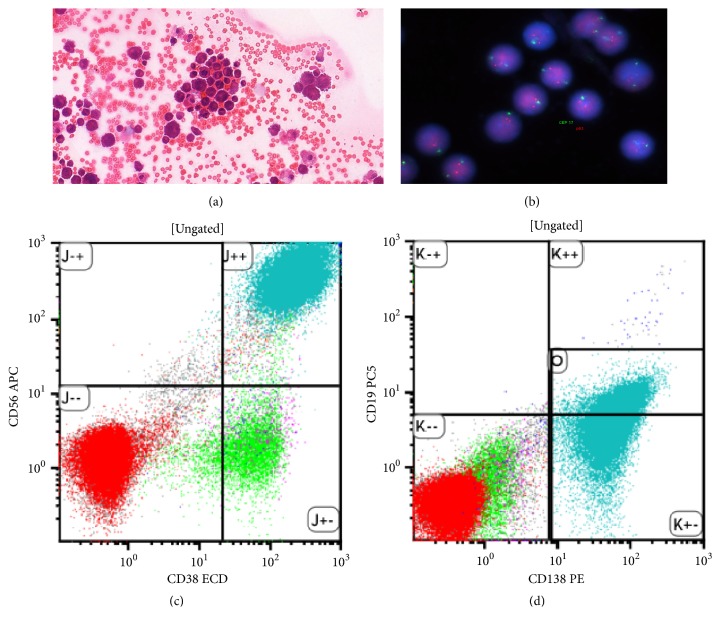
CSF analysis. (a) MGG stained cytospin preparation; (b) fluorescence in situ hybridization (FISH) with probes for 17p (normal); ((c) and (d)) flow cytometry: the plasma cells are CD38, 56, and 138 positive.

To cytologically confirm leptomeningeal disease, lumbar puncture is necessary: in the previously cited review, it was diagnostic in 90% of the cases [6]. Liquor opening pressure is typically raised, and the liquor cell count is elevated. Plasma cells can be cytologically identified (Figure 3(a)). Flow cytometry can confirm monoclonality and aberrant protein expression (Figures 3(c) and 3(d)). CSF protein electrophoresis and free light chain analysis can indirectly confirm the CNS presence of the aberrant plasma cell clone. Fluorescence in situ hybridization (FISH) on the cytospin preparation often shows high-risk features, most commonly deletion 17p (TP53) (Figure 3(b)) [12]. These genetic aberrations contribute to the resistance to traditional chemotherapy and radiotherapy [13].

## 5. Differential Diagnosis

Naturally, there can be many other causes for nervous system symptoms in myeloma patients, making the diagnosis of CNS propagation difficult. Many of the established MM drugs cause peripheral neuropathy. In case of bortezomib, this is usually dominantly sensory, while in case of thalidomide, it is typically a sensomotor neuropathy. Both cause axonal degeneration, and the symptoms are only reversible in case of quick drug interruption/dose reduction; however, if treatment continues, they can become permanent [14]. MM (and other M-protein secretory conditions from MGUS to Waldenström's macroglobulinemia) can cause polyneuropathy on their own right. M-protein is present in 10% of cryptogenic neuropathies, mostly IgM, and in 50% of the IgM cases the M-protein has an anti-myelin associated glycoprotein (anti-MAG) activity [15]. Another complication of the above-mentioned M-protein producing diseases is AL amyloidosis. In case of clinical suspicion, tissue biopsy is needed to confirm the diagnosis of amyloid deposition. The most common biopsy targets are abdominal subcutaneous fat and either rectal mucosal or gingival biopsy.

Other complications, common in MM, such as hyperviscosity, renal failure, and hypercalcemia can also cause CNS symptoms, most commonly disorientation and somnolence. Given the average age of the MM population plus the prothrombotic properties of the IMiDs, vascular events (both ischemia and hemorrhage) can occur. Apart from these, autoimmunity, paraneoplasia, and infections have to be considered (herpes and JC viruses) in these patients with compromised immune system.

Most myeloma patients are taking multiple medications. Among these, thalidomide has a sedative effect, but patients taking thalidomide often report vertigo, tremor, imbalance, and difficulty to walk too. These tend to improve quickly upon stopping thalidomide. Major analgesics (morphine and fentanyl) and adjuvant pain medications (gabapentin, pregabalin, tegretol, and tricyclic antidepressants) can blur the clinical picture further, especially if drug levels are unreliable due to liver and kidney failure.

## 6. Treatment and Survival

The treatment of CNS MM is highly problematic. On one hand, this complication is more common in otherwise high-risk MM (both clinically aggressive and genetically high risk), when chemotherapy is usually less effective. Another problem is that drugs tested and proven in relapsed MM, such as the proteasome inhibitors (bortezomib, carfilzomib, and ixazomib), cannot cross the blood-brain barrier, and standard chemotherapy drugs used to treat CNS lymphoma and brain cancer are not particularly effective in MM. The IMiDs (thalidomide, lenalidomide, and pomalidomide) do cross the blood-brain barrier; however, IMiD resistance is not uncommon in this group of patients. The anti-CD38 antibody daratumumab and anti-CS1 elotuzumab recently approved in relapsed MM were not prospectively tested in this particular group of patients; CNS involvement was an exclusion criterion in the registration trials. It is unknown whether these antibodies can get through the blood-brain barrier, and there is no published report about their intrathecal use, whatever tempting it would be theoretically. In one case report of a CNS MM case, it was found that MM cells in the CSF shred their surface CD38 after starting systemic daratumumab, which was interpreted as an indirect sign of CNS penetration of the drug [16]. It has to be mentioned here that intrathecal injection of bortezomib can be fatal and is therefore absolutely contraindicated!

As a salvage, in analogy to acute lymphoid leukemia, intrathecal combinational chemotherapy with methotrexate, cytosine arabinoside, and dexamethasone (15 mg, 40 mg, and 4 mg, resp.) is often applied continuously until liquor clearance, usually in combination with high-dose chemotherapy, using drugs with known CNS penetration (methotrexate, cytosine arabinoside, idarubicin, and thiotepa), together with high-dose dexamethasone, similar to what we normally do in CNS lymphoma. In case of a response to this strategy, retrospective data support the use of craniocaudal radiotherapy and possibly ASCT as consolidation [7].

Recently, a case review reported the effectivity of pomalidomide in CNS MM [17]. Pomalidomide is a drug of the IMiD class approved for lenalidomide refractory patients, which according to a subgroup analysis is more effective in high-risk cytogenetics, especially deletion 17p, than other IMiDs and has good CNS penetration [18]. Another drug that could have a role in this setting is marizomib, a potent natural second-generation proteasome inhibitor produced by a marine bacterium, which was used successfully in two cases of CNS MM [16].

Based on these data, it is not surprising that the overall survival in this condition is extremely poor. The survival data varies widely between studies but usually does not exceed a couple of months from diagnosis (median survival: 2–8 months, Table 2) [3, 5–11, 19]. It is significantly better, however, in those patients who had craniocaudal irradiation or ASCT. Although this data is biased as these are selected patients who responded to their initial treatment [3, 5], in more recent publications, nevertheless, there is a promising increase in survival [7].

## 7. 13 Cases of CNS MM over 10 Years in Our 2 Centers

**Figure 4:**
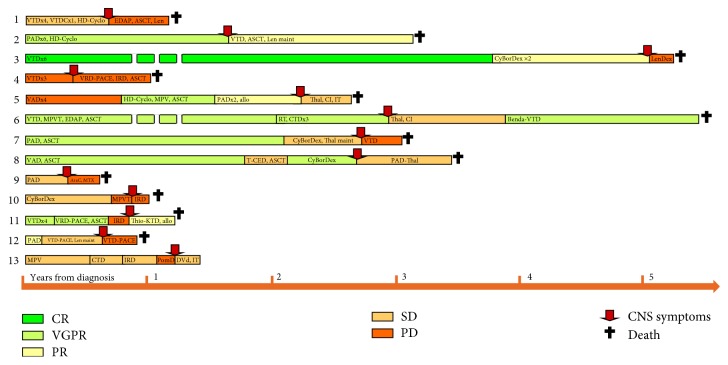
Treatments and responses. Allo: allogeneic stem cell transplantation; amp: amplification; ASCT: autologous stem cell transplantation; Bor: bortezomib; Car: carfilzomib; CI: cranial irradiation; CSF: cerebrospinal fluid; CTD: cyclophosphamide, thalidomide, and dexamethasone; CyBorDex: cyclophosphamide, bortezomib, and dexamethasone; Dara: daratumumab; del: deletion; DVd: daratumumab, bortezomib, and dexamethasone; flow: flow cytometry; hyperd: hyperdiploid; IFX: immunofixation; IRD: ixazomib, revlimid, and dexamethasone; IT: intrathecal chemotherapy; KTD: carfilzomib, thalidomide, and dexamethasone; LC: light chain; Len: lenalidomide; maint: maintenance; MPV: melphalan, prednisolone, and bortezomib; ND: not done; PACE: cisplatin, doxorubicin, cyclophosphamide, and etoposide; PAD: bortezomib, doxorubicin, and dexamethasone; PCL: plasma cell leukemia; PomD: pomalidomide and dexamethasone; RT: radiotherapy; T-CED: thalidomide, cyclophosphamide, etoposide, and dexamethasone; Thal: thalidomide; Thio: thiotepa; VAD: vincristine, doxorubicin, and dexamethasone; VRD: bortezomib, lenalidomide, and dexamethasone; VTD: bortezomib, thalidomide, and dexamethasone.

**Figure 5:**
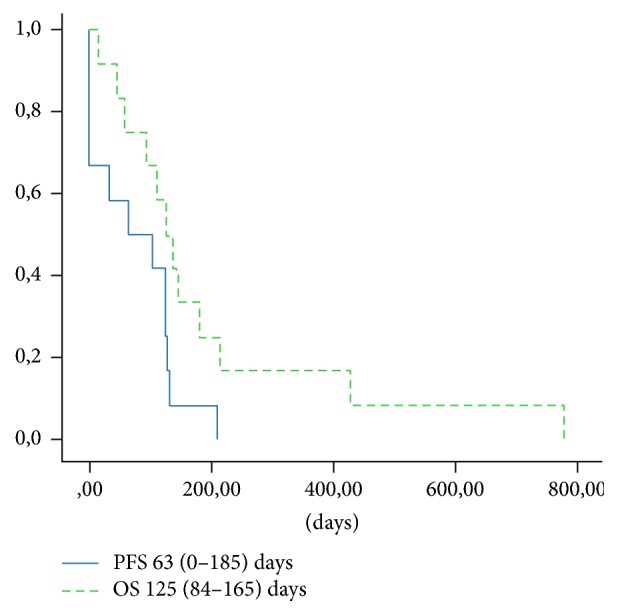
Progression-free survival (PFS) and overall survival (OS).

Out of 548 MM cases treated over 10 years at the 3rd Department of Internal Medicine, Semmelweis University, and the Department of Haematology and the Stem Cell Transplantation, St. István and St. László Hospital, we identified retrospectively 13 cases of CNS MM. Patients' characteristics including symptoms, treatments, and survival are shown in Table 1, treatment courses and responses to various treatment lines are depicted graphically in Figure 4, and progression-free survival and overall survival given in Figure 5.

CNS symptoms started in each case at the time of relapse, on average 2 years from the initial diagnosis of MM (range: 3 months–5.3 years). These patients were younger than the usual MM patients, with more IgA and LC secretory cases than usually seen in MM. In 8/13 cases, high-risk cytogenetics were present at diagnosis, and we observed clonal evolution in terms of new FISH findings in 3 patients. Two patients presented originally as primary plasma cell leukemia; in these cases, survival from the original diagnoses was only 5 and 12 months.

The dominant symptoms were cranial nerve palsies (double vision and dysphagia) but patients also presented with headache, seizures, paraparesis or paraplegia, and radiculopathy. The diagnosis was supported by imaging in 6 and by CSF analysis in 8 cases.

Treatment strategies varied according to the patients' presenting symptoms, performance stage, and drug availability at the time. Ten patients had treatment containing an IMiD (5 lenalidomide and 5 thalidomide), five had high-dose salvage chemotherapy, four ASCT, two craniocaudal irradiation, and six intrathecal chemotherapy. One very recent case (#13) presenting with CNS relapse in September 2017 showed a remarkable response to systemic daratumumab. Daratumumab was combined with bortezomib and high-dose corticosteroids and the patient had 3 times intrathecal triplet chemotherapy treatment as well. With 3-month follow-up, he is alive and well, and his CSF that was previously full of plasma cells (Figure 3(a)) is now clear.

In one case, CNS MM presented as an isolated relapse following allogeneic SCT, indicating that graft versus myeloma effect is less prominent in the CNS. In another case, after combination chemotherapy for CNS MM, we attempted allogeneic SCT with total body irradiation (TBI) based conditioning; this patient died in sepsis but was apparently free of disease.

Survival, in keeping with the literature, was very poor in our series; with the median being 3 months, the longest survival so far was 14 months.

## 8. Concluding Remarks

As in the 13 cases presented in our series, CNS MM is a real challenge to the treating physician. The diagnosis is complex; probably many cases remain undetected. Although there are no clear guidelines regarding best treatment, aggressive management is necessary. Most recently, pomalidomide and marizomib showed promising results, and our cases suggest that monoclonal antibodies, particularly daratumumab, might have a role in this setting, too.

## Figures and Tables

**Table 1:** Patient characteristics.

Patient	At diagnosis					Prior treatment					CNS presentation								Survival (days)			
	Age	Sex	ISS/PCL	FISH/karyotype	M-protein	Lines	Thal	Bor	Len	ASCT	Age	Symptoms	Diagnosis	Systemic relapse	ISS	New FISH	Treatment	Response	OS from CNS	OS from DG	PFS from CNS	DG to CNS
1	69	M	3	1q amp	LC lambda	1	1	1	0	0	69	Double vision	CT, CSF flow	yes	3	ND	EDAP, IT, Mel-ASCT, Len maint	PR	144	335	130	191
2	65	M	1	1q amp	LC kappa	1	0	1	0	0	65	Paraplegia	CSF flow	yes	1	ND	VTD, Mel-ASCT, Len maint	PR	427	897	124	470
3	69	F	1	ND	IgG lambda	2	1	1	0	0	74	Double vision	CSF IFX	no	1	ND	LenDex	NR	56	2025	0	1969
4	41	F	3	1q amp	LC lambda	1	1	1	0	0	41	Headache, hypoglossus, and abducent paresis	Clinical (unspecific MRI abnormalities)	yes	3	17p	VRD-PACE, IT, EDAP, IRD, Mel-Benda-ASCT	PR	180	290	63	110
5	52	M	3	del 13q	IgA kappa	3	1	1	0	Allo	55	Abducent paresis, unable to swallow	CSF cytospin, MRI	no	1	ND	Thal + IT + craniocaudal irrad + discontinue GVHD prophylaxis	CR	134	1103	126	969
6	56	F	3	hyperd	IgA kappa	3	1	1	0	1	60	Headache	MRI	no	1	ND	Thal + craniocaudal irrad, benda-VTD	PR	776	2191	104	1415
7	54	M	3	del 13q	IgA kappa	2	1	1	0	1	56	Oculomotor nerve paresis	Clinical (MRI orbit neg, CSF ND)	yes	3	1qamp	VTD	NR	93	1033	32	940
8	53	M	3	complex, hypodipl	IgG lambda	3	1	1	0	2	57	Abducent paresis	Clinical (MRI neg, CSF ND)	yes	3	same	PAD-Thal	MR	213	1563	210	1350
9	43	M	3, PCL	complex, del 13q	LC lambda	1	0	1	0	0	44	Paresis, cannot swallow	CSF cytospin, MRI: tumors	yes	3	same	HD MTX, AraC, IT	PD	44	140	0	96
10	66	M	3	1q amp	IgG lambda	2	1	1	0	0	67	Abducent paresis	MRI	yes	3	same	IRD	PD	14	386	0	372
11	34	M	2	t(4;14)	IgG lambda	3	1	1	1	1	36	Left leg paresis	MRI, CSF flow	yes	1	same	Thio, Car, Thal, Dex	PR	38	557	38	519
12	60	F	3, PCL	t(11;14), del 17p	IgG kappa	2	1	1	1	1	60	Seizures	CSF cytospin	yes	3	same	VTD-PACE + ith triplet 4x, Mel-TBI allo	PD	110	325	0	215
13	72	M	2	normal	LC kappa	4	1	1	1	0	73	Back pain	CSF flow, CT	yes	ND	1qamp	DVd + IT	PR	60	695	60	675

Mean	56.5			8/12 HR	7/13 lambda	2					58.2								125	897	63	715

Allo: allogeneic stem cell transplantation; amp: amplification; ASCT: autologous stem cell transplantation; Bor: bortezomib; Car: carfilzomib; CI: cranial irradiation; CSF: cerebrospinal fluid; CTD: cyclophosphamide, thalidomide, and dexamethasone; CyBorDex: cyclophosphamide, bortezomib, and dexamethasone; Dara: daratumumab; del: deletion; DVd: daratumumab, bortezomib, and dexamethasone; flow: flow cytometry; hyperd: hyperdiploid; IFX: immunofixation; IRD: ixazomib, revlimid, and dexamethasone; IT: intrathecal chemotherapy; KTD: carfilzomib, thalidomide, and dexamethasone; LC: light chain; Len: lenalidomide; maint: maintenance; MPV: melphalan, prednisolone, and bortezomib; ND: not done; PACE: cisplatin, doxorubicin, cyclophosphamide, and etoposide; PAD: bortezomib, doxorubicin, and dexamethasone; PCL: plasma cell leukemia; PomD: pomalidomide and dexamethasone; RT: radiotherapy; T-CED: thalidomide, cyclophosphamide, etoposide, and dexamethasone; Thal: thalidomide; Thio: thiotepa; VAD: vincristine, doxorubicin, and dexamethasone; VTD: bortezomib, thalidomide, and dexamethasone.

**Table 2:** Largest recently published case series.

	*n*	Era	Median OS (months, CI)	Center(s)	Reference
Jurczyszyn et al.	172	1995–2014	3.7 (NA)	Multicenter: 38 centers from 20 countries	[3]
Paludo et al.	29	1998–2014	3.4 (1–10)	Mayo Clinic, Rochester	[5]
Schluterman et al.	23	1990–2003	3 (0.1–25)	University of Arkansas, Little Rock	[6]
Chen et al.	37	1999–2010	4.6 (2.8–6.7)	Princess Margaret Cancer Centre, Toronto	[7]
Abdallah et al.	35	1996–2012	4 (1–13)	University of Arkansas, Little Rock	[8]
Majd et al.	9	1998–2012	3 (1–12)	Mount Sinai Hospital, New York	[9]
Gozzetti et al.	50	2000–2010	6 (1–23)	GIMEMA (Gruppo Italiano Malattie EMatologiche dell'Adulto) multiple myeloma working party	[10]
Dias et al.	21	2008–2016	5.8 (NA)	Faculdade de Ciências Médicas da Santa Casa de São Paulo	[11]

CI: confidence interval; NA: not available.
